# Inter-arm Blood Pressure Difference is Associated with Recurrent Stroke in Non-cardioembolic Stroke Patients

**DOI:** 10.1038/s41598-019-49294-8

**Published:** 2019-09-04

**Authors:** Yoonkyung Chang, Jinkwon Kim, Yong-Jae Kim, Tae-Jin Song

**Affiliations:** 1 Department of Neurology, Mokdong Hospital, Ewha Womans University College of Medicine, Seoul, Korea; 2 Department of Neurology, Gangnam Severance Hospital, Yonsei University College of Medicine, Seoul, Korea; 3 Department of Neurology, Eunpyeong St. Mary’s Hospital, Catholic University of Korea, Seoul, Korea

**Keywords:** Atherosclerosis, Stroke

## Abstract

Recurrent stroke increases mortality and aggravates the disability of stroke patients. We hypothesized that increased inter-arm systolic blood pressure difference and inter-arm diastolic blood pressure difference would be related to recurrent stroke in non-cardioembolic stroke patients. A total of 1226 consecutive non-cardioembolic first-ever ischemic stroke patients, in whom bilateral brachial blood pressures were measured by an automated ankle-brachial index measuring device, were included in our study. Recurrent stroke was defined as newly developed neurologic symptoms with relevant lesions on brain CT and/or MRI after 7 days or hospital discharge. Inter-arm systolic and diastolic blood pressure differences ≥10 mmHg were noted in 9.7% (120/1226) and 5.0% (62/1226) of patients, respectively. During a median 24 months of follow-up, 105 (8.5%) patients experienced recurrent stroke. Patients who had inter-arm systolic blood pressure difference ≥10 mmHg showed increased risk of recurrent stroke (hazard ratio:1.77, 95% confidence interval: 1.04–3.00, *p* = 0.033). Moreover, inter-arm diastolic blood pressure difference ≥10 mmHg was also independently associated with increased risk of recurrent stroke (hazard ratio:2.92, 95% confidence interval: 1.59–5.34, *p* = 0.001). In conclusion, inter-arm blood pressure difference ≥10 mmHg may be associated with increased risk recurrent stroke in non-cardioembolic stroke patients.

## Introduction

Systolic and diastolic blood pressures are important risk factors for occurrence and recurrence of stroke^1^. The latest guideline for management of hypertension recommended to check blood pressure in both arms^2^, and different result is often found when checked bilaterally. These inter-arm blood pressure differences (IABDs) are reported in general population (4%), diabetic patients (7%) and stroke patients (10%)^3,4^.

Stroke is one of the leading causes of disability, loss of productivity, and poor functional outcome including mortality^5,6^. Disability and poor functional outcome from stroke cause serious burden to patients themselves and their caregivers^6^. Additionally, recurrent stroke accounts for 10–25% of the total stroke^7^. Recurrent stroke increases mortality and exacerbates the disability of stroke patients^8^. Therefore, identifying and modifying factors associated with recurrent stroke are important tasks for treating stroke patients.

IABD can be easily measured at outpatient clinic and can be used as an important indicator or predictor in clinical fields. The major cause of IABD includes atherosclerosis and stenosis due to various diseases in the aorta, subclavian arteries and their branches^9^. In stroke patients, considering the relationship between extensive atherosclerosis and poor clinical outcome^10^, IABD may also have an association with recurrent stroke. However, few studies have reported these issues^11^. Our hypothesis is that increased IABD would be related with recurrent stroke in non-cardioembolic stroke patients.

## Results

### Demographics and comparisons of clinical variables according to IABD ≥10 mmHg

There were no statistical difference of baseline demographics between patients included in the analysis and those excluded, except for age (Supplementary Table 1). Overall, 61.4% (753/1226) were male, and the mean age was 65.0 ± 11.8 years. The ankle-brachial index (ABI) examination was performed at median 4 days [interquartile range 3–6 days]. In total, 148 (12.1%) patients were prescribed antihypertensive medications before performing ABI. Among these 148 patients, 99 (66.9%) had been prescribed intravenous labetalol only, while 23 (15.5%) patients were prescribed intravenous perdipine and labetalol. Patients who received both oral and intravenous antihypertensive agents were 11 (7.4%), and 15 (10.1%) patients were prescribed oral antihypertensive agents only.

The systolic (IASBD) and diastolic (IADBD) blood pressure difference ≥10 mmHg was noted in 9.7% (120/1226) and 5.0% (62/1226), respectively. The patients with IASBD ≥10 mmHg had more frequent history of hypertension, coronary artery disease (CAD), metabolic syndrome, regular alcohol intake, left ventricular hypertrophy, large artery atherosclerosis stroke subtype, cerebral atherosclerosis, and high-grade white matter hyperintensities. Moreover, body mass index, pulse rate, systolic blood pressure, diastolic blood pressure, and baPWV were higher in patients with IASBD ≥10 mmHg compared to those with IASBD <10 mmHg (Table 1). Patients with IADBD ≥10 mmHg had more frequent history of CAD, regular alcohol intake, left ventricular hypertrophy, cerebral atherosclerosis, and high-grade white matter hyperintensities. In addition, body mass index, National Institutes of Health Stroke Scale (NIHSS) score, pulse rate, systolic blood pressure, and diastolic blood pressure were higher in patients with IADBD ≥10 mmHg compared to those with IADBD <10 mmHg (Table 1).

**Table 1:** Clinical characteristics and comparison of study patients with IASBD and IADBD ≥10 mmHg.

	Total (n = 1226)	IASBD <10 mmHg (n = 1109)	IASBD ≥10 mmHg (n = 117)	*p* value	IADBD <10 mmHg (n = 1166)	IADBD ≥10 mmHg (n = 60)	*p* value
Demographics							
Sex (male)	753 (61.4)	683 (61.6)	70 (59.8)	0.710	720 (61.7)	33 (55.0)	0.295
Age, years	65.0 ± 11.8	65.0 ± 11.6	65.2 ± 13.6	0.841	65.0 ± 11.7	65.4 ± 13.3	0.780
Risk factors							
Hypertension	901 (73.5)	807 (72.8)	94 (80.3)	0.078	854 (73.2)	47 (78.3)	0.384
Diabetes mellitus	415 (33.8)	372 (33.5)	43 (36.8)	0.485	398 (34.1)	17 (28.3)	0.354
Hypercholesterolemia	185 (15.1)	163 (14.7)	22 (18.8)	0.238	177 (15.2)	8 (13.3)	0.697
Smoking	321 (26.2)	288 (26.0)	33 (28.2)	0.601	305 (26.2)	16 (26.7)	0.930
Coronary artery disease	250 (20.4)	212 (19.1)	38 (32.5)	0.001	232 (19.9)	18 (30.0)	0.058
Metabolic syndrome	502 (40.9)	440 (39.7)	62 (53.0)	0.005	480 (41.2)	22 (36.7)	0.489
Alcohol intake	140 (11.4)	118 (10.6)	22 (18.8)	0.008	128 (11.0)	12 (20.0)	0.032
Left ventricular hypertrophy	175 (14.3)	136 (12.3)	39 (33.3)	0.001	155 (13.3)	20 (33.3)	0.001
Body mass index, kg/m^2^	24.1 ± 3.0	24.0 ± 2.9	25.6 ± 3.8	0.001	24.1 ± 3.0	25.5 ± 3.6	0.004
Familial history of stroke	325 (26.5)	289 (26.1)	36 (30.8)	0.272	304 (26.1)	21 (35.0)	0.127
Antihypertensive treatment before ABI examination	148 (12.1)	138 (12.4)	10 (8.5)	0.219	142 (12.2)	6 (10.0)	0.613
Thrombolytic therapy	120 (9.8)	104 (9.4)	16 (13.7)	0.137	111 (9.5)	9 (15.0)	0.164
NIHSS	4.0 ± 4.7	4.0 ± 4.5	4.1 ± 4.2	0.714	3.9 ± 4.5	5.1 ± 4.5	0.048
Stroke subtype				0.001			0.001
Large artery atherosclerosis	359 (29.3)	308 (27.8)	51 (43.6)		334 (28.6)	25 (41.7)	
Lacune	357 (29.1)	346 (31.2)	11 (9.4)		352 (30.2)	5 (8.3)	
Undetermined negative	357 (29.1)	321 (28.9)	36 (30.8)		339 (29.1)	18 (30.0)	
Undetermined, two or more causes identified	153 (12.5)	134 (12.1)	19 (16.2)		141 (12.1)	12 (20.0)	
Cerebral atherosclerosis				0.001			0.001
Extracranial atherosclerosis only	179 (14.6)	162 (14.6)	17 (14.5)		170 (14.6)	9 (15.0)	
Intracranial atherosclerosis only	417 (34.0)	375 (33.8)	42 (35.9)		391 (33.5)	26 (43.3)	
Both extra- and intracranial atherosclerosis	136 (11.1)	104 (9.4)	32 (27.4)		118 (10.1)	18 (30.0)	
Previous medication before admission							
Anti-thrombotics	281 (22.9)	261 (23.5)	20 (17.1)	0.115	271 (23.2)	10 (16.7)	0.237
Lipid lowering agents	221 (18.0)	202 (18.2)	19 (16.2)	0.597	212 (18.2)	9 (15.0)	0.532
Discharge medication							
Anti-thrombotics	1201 (98.0)	1086 (97.9)	115 (98.3)	0.791	1144 (98.1)	57 (95.0)	0.119
Lipid lowing agents	1153 (94.0)	1039 (93.7)	114 (97.4)	0.103	1094 (93.8)	59 (98.3)	0.150
Ankle-brachial index parameters							
Pulse rate, per/min	69.3 ± 12.2	69.1 ± 12.0	72.0 ± 3.8	0.029	69.2 ± 12.0	72.5 ± 14.8	0.075
Mean arm SBP, mmHg	149.6 ± 22.5	146.6 ± 20.7	159.3 ± 23.3	0.001	147.1 ± 20.6	160.8 ± 29.7	0.001
Mean arm DBP, mmHg	85.7 ± 12.6	83.4 ± 12.0	90.2 ± 15.3	0.001	83.8 ± 12.0	90.3 ± 18.8	0.010
Mean baPWV, m/s	19.9 ± 4.9	19.5 ± 4.8	20.7 ± 5.9	0.044	19.6 ± 4.9	20.3 ± 4.7	0.240
Mean ABI value	1.1 ± 0.2	1.1 ± 0.3	1.0 ± 0.1	0.654	1.1 ± 0.3	1.1 ± 0.1	0.562
High-grade white matter hyperintensities	287 (23.4)	242 (21.8)	45 (38.5)	0.001	267 (22.9)	20 (33.3)	0.063
Poor functional outcome (mRS >2)	303 (24.7)	253 (22.8)	50 (42.7)	0.001	271 (23.2)	32 (53.3)	0.001

Data are shown as n (%) or mean ± standard deviation.

IASBD: inter-arm systolic blood pressure difference, IADBD: inter-arm diastolic blood pressure difference, NIHSS: National Institutes of Health Stroke Scale, SBP: systolic blood pressure, DBP: diastolic blood pressure, baPWV: brachial-ankle pulse wave velocity, HWHs: high-grade white matter hyperintensities, CMBs: cerebral microbleeds, HPVSs: high-grade perivascular spaces, ALIs: asymptomatic lacunar infarctions.

### Association between IABD and recurrent stroke

During follow-up (median 24 months, interquartile range 14–32 months), 105 (8.6%) patients had experienced recurrent stroke. The recurrent stroke was more frequently demonstrated in the IASBD ≥10 mmHg group compared with the IASBD <10 mmHg group (17.5% vs. 7.5%, p < 0.001) and in the IADBD ≥10 mmHg group compared with the IADBD <10 mmHg group (24.2% vs. 7.7%, p < 0.001), respectively. Considering recurrent stroke subtypes, large artery atherosclerosis (71.4% vs. 28.6%) and two or more causes identified (19% vs. 17.9%) were more frequently noted in the IASBD ≥10 mmHg group compared with the IASBD <10 mmHg group (p = 0.007). Furthermore, hemorrhagic stroke occurred less frequently in the IASBD ≥10 mmHg group compared with the IASBD <10 mmHg group (0.0% vs. 6.0%) (Table 2). For diastolic blood pressure differences, there was no statistical significance between patients with IADBD ≥10 mmHg or IADBD <10 mmHg (Table 2), even though a similar tendency of recurrent stroke subtypes was noted for those with systolic blood pressure differences. Considering the location of recurrent stroke, patients with IASBD ≥10 mmHg had a tendency of recurrent stroke in posterior circulation (61.9% vs. 40.5%, p = 0.077). In contrast, there was no difference in the location of recurrent stroke in patients with IADBD ≥10 mmHg compared to IADBD <10 mmHg. (Recurrent stroke in posterior circulation: 53.3% vs. 43.3%, p = 0.471).

**Table 2:** Subtypes and location of stroke recurrence according to IASBD and IADBD ≥10 mmHg.

	Total (n = 105)	IASBD <10 mmHg (n = 84)	IASBD ≥10 mmHg (n = 21)	*p* value	IADBD <10 mmHg (n = 90)	IADBD ≥10 mmHg (n = 15)	*p* value
Subtypes of recurrent stroke				0.007			0.060
Large artery atherosclerosis	39 (37.1)	24 (28.6)	15 (71.4)		30 (33.3)	9 (60.0)	
Cardioembolism	6 (5.7)	6 (7.1)	0 (0.0)		6 (6.7)	0 (0.0)	
Lacune	14 (13.3)	13 (15.5)	1 (4.8)		14 (15.6)	0 (0.0)	
Undetermined negative	22 (21.0)	21 (25.0)	1 (4.8)		21 (23.3)	1 (6.7)	
Undetermined, two or more causes identified	19 (18.1)	15 (17.9)	4 (19.0)		14 (15.6)	5 (33.3)	
Hemorrhagic stroke	5 (4.8)	5 (6.0)	0 (0.0)		5 (5.6)	0 (0.0)	
Location of stroke recurrence				0.077			0.471
Anterior circulation	58 (55.2)	50 (59.5)	8 (38.1)		51 (56.7)	7 (46.7)	
Posterior circulation	47 (44.8)	34 (40.5)	13 (61.9)		39 (43.3)	8 (53.3)	

Data are shown as n (%) or mean ± standard deviation.

The *p* values are derived by Chi’s square test.

IASBD: inter-arm systolic blood pressure difference, IADBD: inter-arm diastolic blood pressure difference.

**Figure 1:**
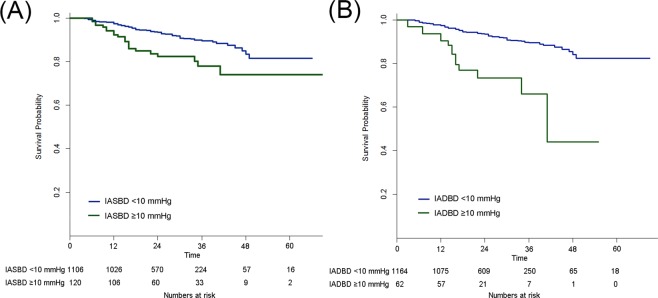
Kaplan–Meier survival plots of recurrent stroke regarding inter-arm systolic blood pressure difference (**A**) and diastolic blood pressure difference (**B**). The Kaplan–Meier curve shows that recurrent stroke depends on inter-arm systolic blood pressure difference (**A**) (p = 0.001) and inter-arm diastolic blood pressure difference (**B**) (p = 0.001).

Kaplan–Meier curves demonstrated that recurrent stroke depended on IASBD ≥10 mmHg (p = 0.001) and IADBD ≥10 mmHg (p = 0.001) (Fig. 1). In multivariate analysis, IASBD ≥10 mmHg was significantly related with recurrent stroke (HR: 1.77, 95% CI: 1.04–3.00, p = 0.033). Furthermore, IADBD ≥10 mmHg was significantly associated with recurrent stroke (HR: 2.92, 95% CI: 1.59–5.34, p = 0.039) (Table 3). Patients with both IASBD and IADBD ≥10 mmHg were also associated with recurrent stroke (HR: 3.02, 95% CI: 1.54–5.91, p = 0.001) (Table 3).

**Table 3:** Frequency of developing clinical events and results of uni and multivariate analysis for recurrent stroke according to IABD.

	Frequency of recurrent stroke		Univariate HR (95% CI)	Multivariate^a^ HR (95% CI)
	Increased IABD group	Reference group		
IASBD				
≥10 mmHg vs. reference (<10 mmHg)	17.5% (21/120)	7.5% (84/1106)	2.26 (1.40–3.65)^*^	1.77 (1.04–3.00)^*^
≥15 mmHg vs. reference (<15 mmHg)	23.8% (15/63)	7.7% (90/1163)	3.20 (1.85–5.54)^*^	1.92 (1.03–3.58)^*^
Absolute difference, mmHg (continuous variable)	N/A	N/A	1.06 (1.03–1.08)^*^	1.04 (1.02–1.07)^*^
IADBD				
≥10 mmHg vs. reference (<10 mmHg)	24.2% (15/62)	7.7% (90/1164)	4.06 (2.35–7.04)^*^	2.92 (1.59–5.34)^*^
≥15 mmHg vs. reference (<15 mmHg)	38.1% (8/21)	8.0% (97/1205)	6.09 (2.95–12.57)^*^	3.61 (1.63–7.99)^*^
Absolute difference, mmHg (continuous variable)	N/A	N/A	1.12 (1.08–1.17)^*^	1.10 (1.05–1.15)^*^
IASBD and IADBD ≥10 mmHg vs. reference (<10 mmHg)	28.2% (11/39)	7.9% (94/1187)	4.40 (2.35–8.22)^*^	3.02 (1.54–5.91)^*^

Cox proportional hazards regression were used for uni- and multivariate analysis.

Data are shown as percent (number of case/numbers of total patients for each group) or hazard ratio (95% confidence interval).

*p < 0.05.

HR: hazard ratio, CI: confidence interval, IABD: inter-arm blood pressure difference, IASBD: inter-arm systolic blood pressure difference, IADBD: inter-arm diastolic blood pressure difference, N/A: not applicable.

a: adjusted for sex, age, hypertension, diabetes mellitus, smoking, coronary artery disease, metabolic syndrome, National Institutes of Health Stroke Scale, stroke subtype, cerebral atherosclerosis, brachial-ankle pulse wave velocity, and high-grade white matter hyperintensities.

In subgroup analysis regarding the relationship of recurrent stroke with IABD, no subgroups showed significant difference except IASBD with increased baPWV (>20.0 m/s) (Supplementary Table 2). The IASBD ≥10 mmHg was associated with recurrent stroke in patients with increased baPWV (>20.0 m/s) (HR: 3.83, 95% CI: 2.20–6.65), (p for interaction = 0.007).

## Discussion

Our study showed that IASBD and/or IADBD ≥10 mmHg was associated with recurrent stroke after adjustment for stroke severity (NIHSS), cerebral atherosclerosis, baPWV, and high-grade white matter hyperintensities, which were closely related factors for stroke. Thus, our study suggests that IASBD or IADBD, which can be easily measured in a clinical field, may be an independent factor for associating or predicting recurrent stroke in non-cardioembolic stroke patients.

Our study demonstrated that IASBD and/or IADBD ≥10 mmHg was associated with recurrent stroke after non-cardioembolic stroke. Previous studies reported that IABD is related with vascular death and all-cause mortality^12^, and these results were consistently noted in populations without known cardiovascular disease^13^ and in chronic kidney disease patients^14^. In elderly patients with hypertension, IASBD ≥10 mmHg was an independent risk factor for increasing the risk of cardiovascular disease and mortality^15^. In contrast, the Framingham Heart Study reported no significant relationship with IABD and mortality^16^. However, up to now, preceding studies regarding IABD and recurrent stroke are rare. In a previous study of patients with acute ischemic stroke, IASBD and/or IADBD ≥10 mmHg was associated with long-term mortality^11^. In patients with non-cardioembolic stroke, cerebral artery stenosis, which is a major predictor for poor prognosis after stroke, was diversely associated with IABD^4^. Another study showed low ABI was associated with recurrent stroke in patients with acute cerebral infarction^17^. Our study supports these findings and may give additive information for the associations of IABD and stroke recurrence. Moreover, bi-brachial blood pressure measurements might play a role as a screening tool for stroke patients to estimate the possibility of recurrent stroke in non-cardioembolic stroke patients.

Our study demonstrated that large artery atherosclerosis stroke subtype was more frequently noted in the IASBD ≥10 mmHg group than in the IASBD <10 mmHg group for the stroke subtype of recurrent stroke. These results are consistent with previous findings that IABD is associated with cerebral atherosclerosis^4^, which is an important risk factor for ischemic stroke occurrence or recurrence. Another study also revealed association of arterial stiffness index and large artery atherosclerosis stroke subtype^18^, and the results of previous studies in which large artery atherosclerosis was associated with asymptomatic lacunar infarction^19^. Meanwhile, hemorrhagic stroke subtype was less frequently noted in the IASBD ≥10 mmHg group than in those with IASBD <10 mmHg. In contrast to our results, previous studies have revealed large artery atherosclerosis, such as aortic atheroma, was significantly associated with cerebral microbleeds that act as imaging biomarkers for future cerebral haemorrhage^19,20^. These results suggest that a large IABD causes cerebral hypoperfusion in the brain, resulting in an ischemic prone state rather than a hemorrhagic prone state, but further research is needed.

Several hypotheses may explain the relationship of IABD with recurrent stroke. IABD is related with advanced atherosclerotic disease in the aorta and its large branches^9^, which may cause an insufficient cerebral blood flow^21^. The hemodynamic dysfunction may be a part of the cause of recurrent stroke or poor clinical outcome^22^. Furthermore, larger atherosclerotic burden is correlated with early poor clinical outcome in stroke population^10^. In addition, previous study discovered that IABD resulting from one-arm ischemia in hypertensive or normotensive patients was related with flow-mediated dilatation^23^, which represents endothelial dysfunction. Increased arterial stiffness may be a link for our study. Arterial stiffness is associated with worse outcome in patients with acute cerebral infarction^24–26^. A population-based study showed large IABD was related with arterial stiffness, which is in line with our study^27^.

There are some limitations in our research. First, although consecutive patients were included in this study, the possibility of selection bias exists because of the retrospective study design. Second, it is difficult to generalize our findings to another population or cohort considering that our study population is limited to a single comprehensive center. Third, multiple, automatic, and simultaneous assessments are recommended for accurate IABD measurements rather than a single, manual, and sequential evaluation methods. We used an automatic and simultaneous measurement device; however, IABD was investigated only once during the ABI assessment and additional follow up data was lacking.

In conclusion, our results demonstrated that IASBD ≥10 mmHg and/or IADBD ≥10 mmHg is associated with recurrent stroke. An IABD ≥10 mmHg could be a useful indicator of risk of recurrent stroke in non-cardioembolic stroke patients.

## Methods

### Study population

Patients admitted to our stroke center between January 2010 and August 2016, with first-ever transient ischemic attack or acute ischemic stroke within 24 hours after symptom onset were enrolled^28^. For total 1782 patients, medical history, demographics, previous history of cardiovascular risk factors, neuroimaging findings and neurologic examination data were collected. All admitted stroke patients underwent chest x-ray, routine blood tests, and electrocardiography. Brain computed tomography (CT) and/or magnetic resonance imaging (MRI) and vascular imaging with MRI or CT were performed. In our department, ABI examination was investigated as one of the routine procedures to evaluate peripheral arterial occlusive lesions, performed at subacute stage (3–7 days after admission)^29^. Our study was approved by Ewha Womans University Mokdong hospital Institutional Review Board (IRB number 2017–04–017), and the requirement of obtaining patients’ informed consent was waived because of the retrospective, cross-sectional, and observational nature of the study. All research was performed in accordance with relevant guidelines and regulations.

Among the 1782 patients, those with potential cardiac source of embolism (PCSE; n = 261, which including persistent atrial fibrillation (AF)/flutter (n = 190), paroxysmal AF (n = 45), sick sinus syndrome (n = 8), and other PCSE (n = 18)), stroke subtype of other determined (rare causes)(n = 23), stroke subtype of undetermined incomplete evaluation (n = 12) and transient ischemic attack (n = 141) were not included. Patients with PCSE were not included because AF can cause inaccurate measurements of brachial-ankle pulse wave velocity (baPWV) and blood pressures (systolic and diastolic)^4,30^. Patients who did not perform brain MRI (n = 22) or with poor image quality (n = 6) or with missing ABI data (n = 43) were excluded from this study. Patients having abnormal ABI (less than 0.9, n = 48) were also excluded because baPWV results could be checked inaccurately^31^. The final number of subjects were 1,226 (Supplementary Fig. 1). Definitions for vascular risk factors are described in the supplemental methods and in a prior study^4,32^. Stroke was classified with the Trial of Org 10172 in Acute Stroke Treatment classification system^33^. Neurological severity was investigated using the NIHSS score. Antihypertensive treatment before ABI was defined as in case of treatment with intravenous or oral antihypertensive agents were performed before ABI examination was undertaken.

### Measuring blood pressure in both arms and inter-arm blood pressure differences

Details for measurement of ABI were described in previous study^34^. In brief, The ABI test was performed by a well-trained examiner with more than 5 years of experience. Before taking the exam, patients had at least 5 minutes rest in a quiet room. The bilateral brachial systolic and diastolic blood pressures were measured automatically and simultaneously with an automated device for ABI test (VP-1000; Colin Co. Ltd, Komaki, Japan) in supine position after discharge of the bowels/bladder in the morning. Pressure cuffs were wrapped on both the brachial and posterior tibial arteries to measure the arterial blood pressure using the oscillometric method^34^. The ABI test was performed after the patients became neurologically stable. A large systolic (IASBD) or diastolic IABD (IADBD) was defined as an absolute inter-arm blood pressure difference ≥10 mmHg, which is frequently used and validated for major cardiovascular outcome as a cut-off value^3,35^.

### Cerebral atherosclerosis and high-grade white matter hyperintensities

The degree of intracranial and extracranial cerebral atherosclerosis (ICAS and ECAS) stenosis was measured using brain CT angiography, MR angiography and/or digital subtraction angiography^19^. The existence of arterial stenosis was defined as more than 50% reduction in luminal diameters^36,37^. The Fazekas score of ≥2 in the deep or periventricular white matter on T2-weighted image or fluid-attenuated inversion recovery were defined as high-grade white matter hyperintensities^38^.

### Outcome measures

The follow up schedule for the patients was three months, one year, and every year after discharge. In each follow up, vital signs, newly developed vascular risk factors, and recurrence of stroke were assessed by a well-trained stroke nurse and/or stroke specialist. If a patient could not make routine follow up, clinical data was obtained by telephone interview. Recurrent stroke was defined as newly developed neurologic symptoms with relevant lesions on brain CT and/or MRI after 7 days after an index stroke or hospital discharge. We estimated time from admission to the first recurrent stroke^28^.

### Statistical analysis

For statistical analysis, we used R package (version 3.0.2; R Foundation for Statistical Computing, Vienna, Austria) and SPSS package (version 23.0, Chicago, IL, USA) programs for Windows. To compare groups according to the presence of IABD ≥10 mmHg, independent t-test, Mann–Whitney U test, Chi-square test, and Fisher’s exact test were performed. To investigate the association of IABD with recurrent stroke, the Kaplan–Meier estimator, log-rank test and Cox proportional hazards regression analysis were used. In multivariate analyses, age, sex and variables with *p* < 0.1 in univariate analysis were included. For sensitivity analysis, IASBD ≥15 mmHg, IADBD ≥15 mmHg, and any increased IABD (systolic or diastolic) were also investigated^4^. Subgroup analyses included sex, age, body mass index, cerebral atherosclerosis, stroke subtype (non-lacunar stroke vs. lacunar stroke subtype) and baPWV. The p value of less than 0.05 by two-tailed was considered as having statistical significance.

## Supplementary information

Supplementary file

## Data Availability

The data that support the findings of this study are openly available in *figshare* at 10.6084/m9.figshare.7542770.

## References

[1] Hong KS (2017). Blood Pressure Management for Stroke Prevention and in Acute Stroke. J Stroke.

[2] Whelton PK (2018). 2017 ACC/AHA/AAPA/ABC/ACPM/AGS/APhA/ASH/ASPC/NMA/PCNA Guideline for the Prevention, Detection, Evaluation, and Management of High Blood Pressure in Adults: A Report of the American College of Cardiology/American Heart Association Task Force on Clinical Practice Guidelines. Hypertension.

[3] Clark CE, Taylor RS, Shore AC, Campbell JL (2016). Prevalence of systolic inter-arm differences in blood pressure for different primary care populations: systematic review and meta-analysis. Br J Gen Pract.

[4] Song TJ (2017). Is obstructive sleep apnea associated with the presence of intracranial cerebral atherosclerosis?. Sleep Breath.

[5] Roger VL (2011). Heart disease and stroke statistics—2011 update: a report from the American Heart Association. Circulation.

[6] Shubhakaran KP, Chin JH (2015). The global burden of neurologic diseases. Neurology.

[7] Kolominsky-Rabas PL (1998). A prospective community-based study of stroke in Germany—the Erlangen Stroke Project (ESPro): incidence and case fatality at 1, 3, and 12 months. Stroke.

[8] Wang A (2016). Effect of recurrent stroke on poor functional outcome in transient ischemic attack or minor stroke. Int J Stroke.

[9] Clark CE, Aboyans V (2015). Interarm blood pressure difference: more than an epiphenomenon. Nephrol Dial Transplant.

[10] Roquer J (2007). Atherosclerotic burden and early mortality in acute ischemic stroke. Arch Neurol.

[11] Kim J (2013). Interarm blood pressure difference and mortality in patients with acute ischemic stroke. Neurology.

[12] Clark CE, Taylor RS, Shore AC, Ukoumunne OC, Campbell JL (2012). Association of a difference in systolic blood pressure between arms with vascular disease and mortality: a systematic review and meta-analysis. Lancet.

[13] Aboyans V (2007). The vital prognosis of subclavian stenosis. J Am Coll Cardiol.

[14] Agarwal R, Bunaye Z, Bekele DM (2008). Prognostic significance of between-arm blood pressure differences. Hypertension.

[15] Clark CE, Taylor RS, Shore AC, Campbell JL (2012). The difference in blood pressure readings between arms and survival: primary care cohort study. Bmj.

[16] Weinberg I, Gona P, O'Donnell CJ, Jaff MR, Murabito JM (2014). The systolic blood pressure difference between arms and cardiovascular disease in the Framingham Heart Study. Am J Med.

[17] Tsivgoulis G (2012). Low ankle-brachial index predicts early risk of recurrent stroke in patients with acute cerebral ischemia. Atherosclerosis.

[18] Tuttolomondo A (2017). Endothelial function and arterial stiffness indexes in subjects with acute ischemic stroke: Relationship with TOAST subtype. Atherosclerosis.

[19] Song TJ (2016). Association between Aortic Atheroma and Cerebral Small Vessel Disease in Patients with Ischemic Stroke. J Stroke.

[20] Song TJ (2014). Association of cerebral microbleeds with mortality in stroke patients having atrial fibrillation. Neurology.

[21] Ochoa VM, Yeghiazarians Y (2011). Subclavian artery stenosis: a review for the vascular medicine practitioner. Vasc Med.

[22] Caplan LR, Wong KS, Gao S, Hennerici MG (2006). Is hypoperfusion an important cause of strokes? If so, how?. Cerebrovasc Dis.

[23] Hu W (2014). The inter-arm diastolic blood pressure difference induced by one arm ischemia: a new approach to assess vascular endothelia function. PLoS One.

[24] Tabata N (2017). Relationship between asymptomatic intra-cranial lesions and brachial-ankle pulse wave velocity in coronary artery disease patients without stroke. Hypertens Res.

[25] Kim J (2014). Brachial-ankle pulse wave velocity for predicting functional outcome in acute stroke. Stroke.

[26] Kim J (2014). Brachial-ankle pulse wave velocity is a strong predictor for mortality in patients with acute stroke. Hypertension.

[27] Canepa M (2013). Relationship between inter-arm difference in systolic blood pressure and arterial stiffness in community-dwelling older adults. J Clin Hypertens (Greenwich).

[28] Song TJ (2015). Low Plasma Proportion of Omega 3-Polyunsaturated Fatty Acids Predicts Poor Outcome in Acute Non-Cardiogenic Ischemic Stroke Patients. J Stroke.

[29] Chung JW (2015). Blood pressure variability and the development of early neurological deterioration following acute ischemic stroke. J Hypertens.

[30] Stergiou GS, Kollias A, Destounis A, Tzamouranis D (2012). Automated blood pressure measurement in atrial fibrillation: a systematic review and meta-analysis. J Hypertens.

[31] Motobe K (2005). Cut-off value of the ankle-brachial pressure index at which the accuracy of brachial-ankle pulse wave velocity measurement is diminished. Circ J.

[32] Song TJ, Chang Y, Shin MJ, Heo JH, Kim YJ (2015). Low levels of plasma omega 3-polyunsaturated fatty acids are associated with cerebral small vessel diseases in acute ischemic stroke patients. Nutr Res.

[33] Adams HP (1993). Classification of subtype of acute ischemic stroke. Definitions for use in a multicenter clinical trial. TOAST. Trial of Org 10172 in Acute Stroke Treatment. Stroke.

[34] Yamashina A (2002). Validity, reproducibility, and clinical significance of noninvasive brachial-ankle pulse wave velocity measurement. Hypertens Res.

[35] Verberk WJ, Kessels AG, Thien T (2011). Blood pressure measurement method and inter-arm differences: a meta-analysis. Am J Hypertens.

[36] Samuels OB, Joseph GJ, Lynn MJ, Smith HA, Chimowitz MI (2000). A standardized method for measuring intracranial arterial stenosis. AJNR Am J Neuroradiol.

[37] Barnett HJM (1991). Beneficial effect of carotid endarterectomy in symptomatic patients with high-grade carotid stenosis. N Engl J Med.

[38] Fazekas F, Chawluk JB, Alavi A, Hurtig HI, Zimmerman RA (1987). MR signal abnormalities at 1.5 T in Alzheimer's dementia and normal aging. AJR Am J Roentgenol.

