# Supplementary material for: Inter-arm Blood Pressure Difference is Associated with Recurrent Stroke in Non-cardioembolic Stroke Patients
Source: Sci Rep. 2019 Sep 4;9:12758. doi: 10.1038/s41598-019-49294-8 (PMC6726617; doi:10.1038/s41598-019-49294-8)
Supplement: Supplementary file 1 — Supplementary file [file 41598_2019_49294_MOESM1_ESM.doc]

**Title:** **Inter-arm Blood Pressure Difference is Associated with Recurrent Stroke in Non-cardioembolic Stroke Patients**

**Authors**: Yoonkyung Chang, MD^1^, Jinkwon Kim, MD, PhD^2^, Yong-Jae Kim, MD, PhD^3^, Tae-Jin Song, MD, PhD^1^

^1^Department of Neurology, Mokdong Hospital, Ewha Womans University College of Medicine, Korea

^2^Department of Neurology, Gangnam Severance Hospital, Yonsei University College of Medicine, Seoul, Korea

^3^Department of Neurology, Eunpyeong St. Mary’s Hospital, Catholic University of Korea, Seoul, Korea

**Address correspondence and reprint requests to:**

Tae-Jin Song, MD, PhD. Department of Neurology, Mokdong Hospital, Ewha Womans University College of Medicine, 1071 Anyangcheon-ro, Yangcheon-gu, Seoul, 07985, Korea

Tel: +82-2-2650-2677, Fax: +82-2-2650-5958; E-mail: knstar@ewha.ac.kr

**Supplementary figure S1.** Flowchart of participants according to inclusion and exclusion criteria


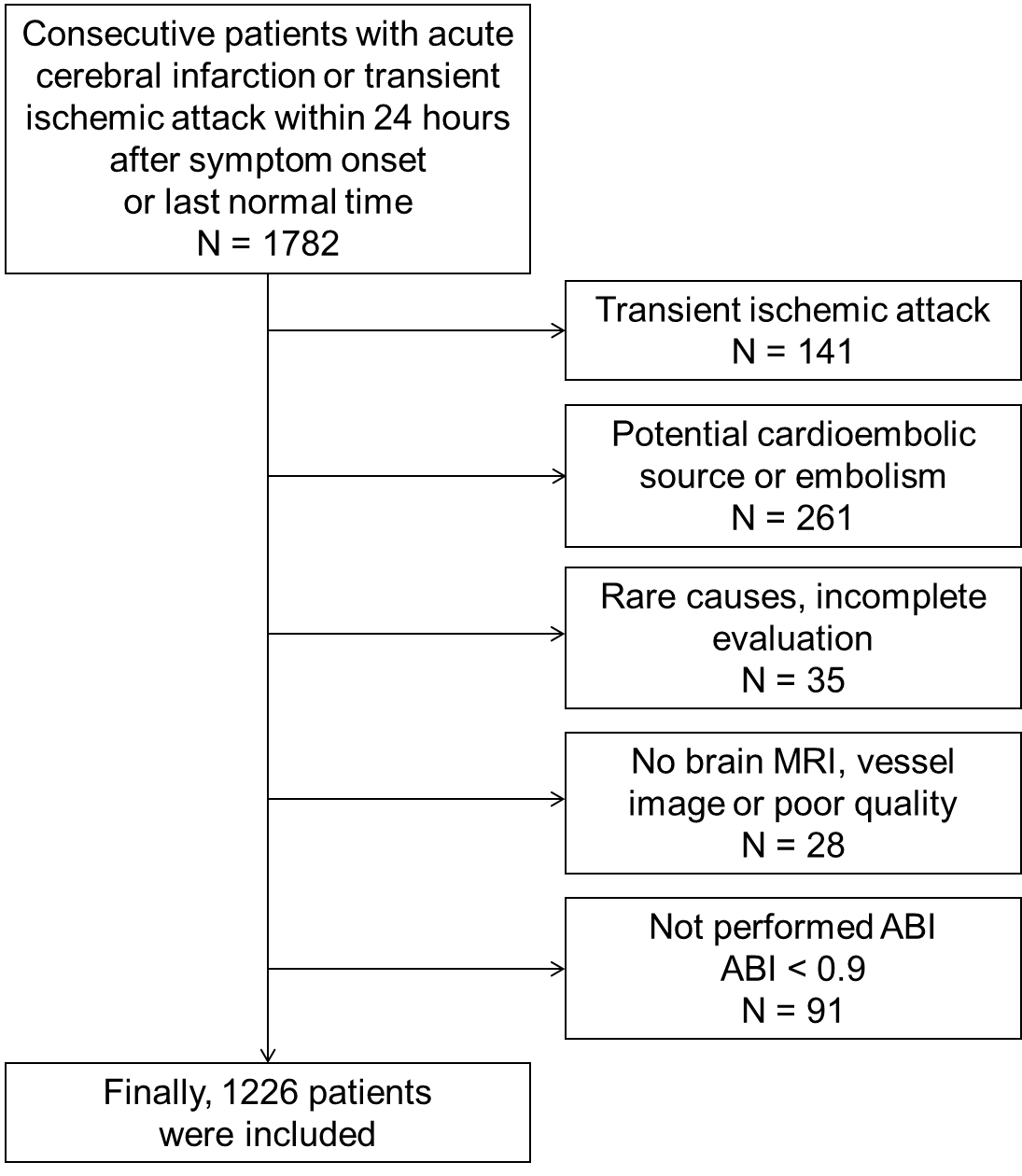


**Supplementary Methods**

***Clinical variables***

A detailed definition of risk factors for hypertension was described in a previous study [1]. In brief, hypertension was defined as being present when a patient had been taking blood pressure-lowering agents, or had a resting systolic blood pressure ≥140 mmHg or diastolic blood pressure ≥90 mmHg on repeated measurements. Diabetes mellitus was diagnosed when the patient had a fasting blood glucose level ≥7.0 mmol/L, or was being treated with oral glucose-lowering medications or insulin. Hyperlipidemia was diagnosed if the patient had total cholesterol ≥6.2 mmol/L, low-density lipoprotein cholesterol ≥4.1 mmol/L, or if the patient had taken lipid-lowering medications after a diagnosis of hyperlipidemia. Patients were defined as smokers if they were current smokers or had stopped smoking within 1 year before the index stroke. Subjects whose recent mean weekly alcohol intake had regularly exceeded 300 g of ethanol were classified as heavy drinkers [2]. Coronary artery disease was defined as a history of myocardial infarction, unstable angina, or angiographically confirmed coronary artery occlusive disease. Metabolic syndrome was diagnosed when a patient had more than three components including abdominal obesity (waist circumference >102 cm for men, >88 cm for women); triglycerides ≥1.7 mmol/L; low level of high-density lipoprotein (<1.04 mmol/L for men, <1.30 mmol/L for women); or blood pressure greater than 130/85 mmHg; or fasting glucose ≥6.1 mmol/L. Left ventricular hypertrophy was diagnosed when electrocardiography findings were matched with at least one of the Sokolow-Lyon index, the Romhilt-Estes point score system, and the Cornell voltage criteria [3].

**References**

1. Song, T. J. *et al.* Is obstructive sleep apnea associated with the presence of intracranial cerebral atherosclerosis? *Sleep Breath* **21**, 639-646, doi: 10.1007/s11325-016-1450-9 (2017)

2. Song, T. J. *et al.* Association between aortic atheroma and cerebral small vessel disease in patients with ischemic stroke. *J Stroke* **18**, 312-320 (2016)

3. Schillaci, G. *et al.* Improved electrocardiographic diagnosis of left ventricular hypertrophy. *Am J Cardiol* **74**, 714–719 (1994)

**Supplementary table 1. Comparison of acute stroke patients during study period who included and excluded for this study**

|  | **Included**  **(n = 1226)** | **Not included**  **(n = 556)** | ***p* value** |
| --- | --- | --- | --- |
| Demographic characteristics |  |  |  |
| Sex (male) | 753 (61.4) | 342 (61.5) | 0.971 |
| Age, years | 65.0 ± 11.8 | 69.5 ± 11.7 | 0.025 |
| Body mass index, kg/m^2^ | 24.1 ± 3.0 | 24.3 ± 3.2 | 0.361 |
| Risk factors |  |  |  |
| Hypertension | 901 (73.5) | 421 (75.7) | 0.319 |
| Diabetes mellitus | 415 (33.8) | 200 (36.0) | 0.383 |
| Hypercholesterolemia | 185 (15.1) | 100 (18.0) | 0.122 |
| Smoking | 321 (26.2) | 148 (26.6) | 0.846 |
| Coronary artery disease | 250 (20.4) | 132 (23.7) | 0.110 |
| Metabolic syndrome | 502 (40.9) | 233 (41.9) | 0.703 |
| Alcohol intake | 140 (11.4) | 50 (9.0) | 0.124 |
| Left ventricular hypertrophy | 175 (14.3) | 76 (13.7) | 0.734 |

Data are shown as n (%), mean ± standard deviation.

**Supplementary Table 2.** Subgroup analysis for recurrent stroke.

|  | **IASBD ≥10 mmHg** | | | **p for interaction** | **IADBD ≥10 mmHg** | | | **p for interaction** |
| --- | --- | --- | --- | --- | --- | --- | --- | --- |
|  | HR | 95% CI | |  | HR | 95% CI | |  |
| Sex, male | 3.98 | 1.62 | 9.77 | 0.462 | 3.78 | 1.94 | 7.35 | 0.491 |
| Sex, female | 1.83 | 1.03 | 3.26 |  | 5.60 | 2.06 | 15.18 |  |
| Age <65 | 1.27 | 0.49 | 3.27 | 0.125 | 2.03 | 0.62 | 6.60 | 0.159 |
| Age ≥65 | 3.00 | 1.70 | 5.29 |  | 5.56 | 2.94 | 10.51 |  |
| Body mass index <24 | 2.08 | 0.88 | 4.91 | 0.804 | 1.94 | 0.60 | 6.28 | 0.117 |
| Body mass index ≥24 | 2.32 | 1.28 | 4.20 |  | 5.56 | 2.94 | 10.54 |  |
| Cerebral atherosclerosis (-) | 1.17 | 0.15 | 9.04 | 0.721 | 7.40 | 0.94 | 57.89 | 0.474 |
| Cerebral atherosclerosis (+) | 1.92 | 1.17 | 3.16 |  | 2.91 | 1.64 | 5.16 |  |
| Non-lacunar stroke subtype | 2.29 | 1.41 | 3.72 | 0.885 | 4.19 | 2.41 | 7.27 | 0.897 |
| Lacunar stroke subtype | 0.04 | 0.00 | N/A |  | 0.04 | 0.00 | N/A |  |
| baPWV ≤20.0 m/s | 0.46 | 0.11 | 1.92 | 0.007 | 0.04 | 0.00 | 1358.08 | 0.898 |
| baPWV >20.0 m/s | 3.83 | 2.20 | 6.65 |  | 5.23 | 2.89 | 9.46 |  |

Values are presented as HR (95% CI).

IASBD: inter-arm systolic blood pressure difference, IADBD: inter-arm diastolic blood pressure difference, HR: hazard ratio, CI: confidence interval, baPWV: brachial ankle pulse wave velocity.
